# Useful Dual Functional of Entropic Information Measures

**DOI:** 10.3390/e22040491

**Published:** 2020-04-24

**Authors:** Angelo Plastino, Mario Carlos Rocca, Flavia Pennini

**Affiliations:** 1 Consejo Nacional de Investigaciones Científicas y Tecnológicas, (IFLP-CCT-CONICET)-C. C. 727, 1900 La Plata, Argentina; plastino@fisica.unlp.edu.ar (A.P.); rocca@fisica.unlp.edu.ar (M.C.R.); 2 Departamento de Física, Universidad Nacional de La Plata, 1900 La Plata, Argentina; 3 Departamento de Matemática, Universidad Nacional de La Plata, 1900 La Plata, Argentina; 4 Fac. de C. Exactas-National University La Pampa, Peru y Uruguay, Santa Rosa, 6300 La Pampa, Argentina; 5 Departamento de Física, Universidad Católica del Norte, Av. Angamos 0610, 2340000 Antofagasta, Chile

**Keywords:** entropic functionals, coherent states, pure-state associated entropies

## Abstract

There are entropic functionals galore, but not simple objective measures to distinguish between them. We remedy this situation here by appeal to Born’s proposal, of almost a hundred years ago, that the square modulus of any wave function ${\left|\psi \right|}^{2}$ be regarded as a probability distribution *P*. the usefulness of using information measures like Shannon’s in this pure-state context has been highlighted in [*Phys. Lett. A***1993**, *181*, 446]. Here we will apply the notion with the purpose of generating a dual functional [$F_{\alpha_{R}}:\left\{S_{Q}\right\}⟶R^{+}$], which maps entropic functionals onto positive real numbers. In such an endeavor, we use as standard ingredients the coherent states of the harmonic oscillator (CHO), which are unique in the sense of possessing minimum uncertainty. This use is greatly facilitated by the fact that the CHO can be given analytic, compact closed form as shown in [*Rev. Mex. Fis. E* **2019**, *65*, 191]. Rewarding insights are to be obtained regarding the comparison between several standard entropic measures.

## 1. Introduction

Dual functionals map ordinary functionals onto real numbers. We are here interested in entropic functionals (EF). There are EFs galore, but no simple objective measures to distinguish between them. We remedy this situation in this work by appealing to Born’s proposal, of almost a hundred years ago, that the square modulus of any wave function ${\left|\psi \right|}^{2}$ ought to be regarded as a probability distribution *P*.

We begin by reminding the reader that the notion of appealing to just a small quantity of expectation values so as to describe main features of physical systems underlies statistical mechanics, particularly in its information theory version, called MaxEnt by its creator (Jaynes). Indeed, theoretical developments of the last century led Jaynes to formulate his MaxEnt approach, which is known to yield the least biased representation consistent with the available data-amount [1,2,3,4,5,6,7,8].

For a similar, but purely quantum treatment in the style of Born, so as to describe pure quantum states, important advances were made in References [9,10,11,12,13,14,15,16], in which a “quantum entropy functional” $S_{Q}$ was utilized, and the MaxEnt approach profitably employed. As an aside, we mention that, precisely, *the MaxEnt approach has become the main comparison-via, till now, to ascertain whether a certain entropic measure is more or less apt than another in describing a given scientific phenomenon*.

Returning to the pure-states entropic measure $S_{Q}\;;\;Q=\left(q_{1},q_{2},\ldots ,q_{n}\right)$, the MaxEnt methodology was demonstrated to be very useful in describing both ground and excited states of variegated many-body problems [9,10,11,12,13]. It constituted a reasonable alternative to the celebrated Gutzwiller ansatz [15], and paved the way for rather interesting semi-classical treatments [16]. It has been shown to provide one with many-body wave functions of a better quality in several distinct scenarios, like the Hartree-Fock [10], the BCS [11], or the random phase approximation [13] ones. One appeals there to a Shannon’s logarithmic ignorance measure [4] for the probability distribution $P_{i}$,

(1) $$S\left[P\right]=-\sum_{i}P_{i}\ln\left(P_{i}\right),$$

with a special choice for the probability distribution (PD)

(2) $$S\left(\psi \right)=-2\sum_{i}{\left|c_{i}\right|}^{2}\ln\left[|c_{i}|\right]$$

for, in an arbitrary basis |i>,

(3) $$\psi =\sum_{j}c_{j}|j>$$

in self explanatory notation.

### The Quantum Entropic Functional $S_{Q}$

Several important properties of the quantum entropy $S_{Q}$ were demonstrated in Reference [16], namely:

- $S_{Q}$ is a true ignorance function, in the sense of Brillouin. For a normalized, discrete probability distribution $p_{i}$, for instance, Shannon’s measure represents the missing information that one would need to possess so as to be in a “complete information” situation (CIS). In a CIS, just one $p_{i}=1$, while the remaining ones vanish [4,5].
- There is a unique global minimum for $S_{Q}$ subject to appropriate MaxEnt constraints.
- $S_{Q}$ obeys an H-theorem.
- Ground state wave functions that maximize $S_{Q}$ satisfy the virial theorem and the hyper virial ones [17].

We see then that our ignorance measure [4] $S_{Q}$ exhibits adequate credentials to be seriously considered. the wave function (wf) we will be interested in here is that advanced in References [18,19], which compactly describes in simple analytic terms the coherent states of the harmonic oscillator (HO), advantageously replacing the usual, cumbersome infinite sum.

## 2. A Recently Developed Analytic, Compact Expression for Coherent States

Reference [18] introduced for the first time ever an analytic, compact expression for coherent states, that was a posteriori extensively discussed in Reference [19]. the *new* coherent states’ compact expression advantageously replaces the customary Glauber’s infinite expansion in terms of the harmonic oscillator eigenstates |n>. It reads

(4) $$\psi_{\alpha }\left(x\right)={\left(\frac{mw}{\pi \hbar }\right)}^{\frac{1}{4}}e^{-\frac{\alpha^{2}}{2}}e^{-\frac{{\left|\alpha \right|}^{2}}{2}}e^{-\frac{mwx^{2}}{2\hbar }}e^{\sqrt{\frac{2mw}{\hbar }}\alpha x}.$$

These $\psi_{\alpha }\left(x\right)$ are eigenfunctions of the annihilation operator *a* corresponding to the one dimensional HO. Thus,

(5) $$|\alpha >={\left(\frac{mw}{\pi \hbar }\right)}^{\frac{1}{4}}e^{-\frac{\alpha^{2}}{2}}e^{-\frac{{\left|\alpha \right|}^{2}}{2}}\int e^{-\frac{mwx^{2}}{2\hbar }}e^{\sqrt{\frac{2mw}{\hbar }}\alpha x}|x>dx.$$

and

(6) a|α>=α|α>.

For α=0 we have

(7) $$\psi_{0}\left(x\right)={\left(\frac{mw}{\pi \hbar }\right)}^{\frac{1}{4}}e^{-\frac{mwx^{2}}{2\hbar }}=\phi_{0}\left(x\right),$$

namely, the wave function (wf) for the HO-ground state, which is a coherent state itself. For simplicity, in what follows we set

(8) $$\frac{mw}{\hbar }=1.$$

Given a certain operator *A*, it is certainly much easier to compute <α|A|α> (just one integral) than an infinite number of <n|A|n> (for *n* phonons) and then sum over them.

Our $\psi_{\alpha }\left(x\right)$, eigenfunctions of the annihilation operator *a* corresponding to the one dimensional HO, exhibit a special property that is of the essence for our present purposes: they are states of minimum Heisenberg-uncertainty. Actually, this is their principal feature, to such an extent that it becomes its defining trait: a coherent state is that of minimum uncertainty (with regards to canonical conjugate variables). This translates into the fact that their associated quantal entropy $S_{Q}$, a measure of ignorance [4], is unique in the sense that the associated quantum ignorance is minimal.

Our central proposal here emerges in this context—*associate to any entropic functional $S_{Q}\left(P\right)$ a numerical real value. This value emerges when the P input of $S_{Q}$ is a coherent state*. This idea is viable because, as we will see, this functional’s numerical associated value *m* is independent of the displacement factor α of the coherent state. *m* is the same for any arbitrary α and thus uniquely characterizes any arbitrary dual functional ${F[S_{Q}]}_{\alpha_{R}}$

(9) $$F_{\alpha_{R}}:\left\{S_{Q}\right\}⟶R^{+}.$$

## 3. Some Different Monoparametric Ignorance Measures

Shannon’s logarithmic measure (1) does not possess any parameter. Generalized entropic measures (GEMs) do [the best summary for them is, in our view, Reference [20] (and references therein). They have become quite popular in the last 30 years, being applied to variegated scientific areas of endeavor, from high energy physics to Economics. There are many GEMs [21], but we will limit ourselves in this Section to four monoparametric ones.

Let F(x) be the probability density (PD) corresponding to a wave function ψ(x), of the form

(10) $$F\left(x\right)=\psi^{*}\left(x\right)\psi \left(x\right).$$

Shannon’s entropic measure (or ignorance measure) is (we set Boltzmann’s constant $k_{B}=1$)

(11) $$S_{S}=-\int F\left(x\right)\ln\left[F\left(x\right)\right]dx.$$

Tsallis’ ignorance measure reads [20]

(12) $$S_{Tq}=-\frac{1-\int {\left[F\left(x\right)\right]}^{q}dx}{q-1},$$

while Rènyi’s one adopts the appearance [20]

(13) $$S_{Rq}=-\frac{1}{1-q}\ln\left\{\int {\left[F\left(x\right)\right]}^{q}dx\right\}.$$

Finally, Kaniadakis’ ignorance measure is [22,23,24]

(14) $$S_{Kq}=-\frac{1}{2q}\left\{\int {\left[F\left(x\right)\right]}^{1+q}dx-\int {\left[F\left(x\right)\right]}^{1-q}dx\right\}.$$

## 4. The  Main Mathematical Tool of This Paper

The coherent state PD is, for complex α,

(15) $$\alpha =\alpha_{R}+i\alpha_{I},$$

given by

(16) $$F_{\alpha }\left(x\right)=<\psi_{\alpha }\left(x\right)|\psi_{\alpha }\left(x\right)>=\pi^{-\frac{1}{2}}e^{-{\left(x-\sqrt{2}\alpha_{R}\right)}^{2}}=F_{\alpha_{R}}\left(x\right)$$

and obviously depends only on the real part $\alpha_{R}$ of α.

Given the probability density *F* for our coherent state, our fundamental tool is to be introduced at this point, via the formal introduction of a dual functional F of a given ignorance measure S(F) (*S* is a functional of *F*). In practice, however, to evaluate F we just compute the functional S(F)

(17) $$F_{\alpha_{R}}\left(S\right)=S\left(F_{\alpha_{R}}\right).$$

We apply it now to our current five ignorance measures, beginning with Shannon’s $S_{S}$

(18) $$F_{\alpha_{R}}\left(S_{S}\right)=S_{S}\left(F_{\alpha_{R}}\right)=\frac{1}{2}\left(1+\ln\pi \right)∼1.07,$$

which is independent of $\alpha_{R}$! This feature is common to all of our five measures, and can be generalized to other generalized measures.

### 4.1. Important Comment on the Meaning of Equation (18)

Let us consider now the specific real number associated with Shannon’s measure

(19) $$N_{S}=\frac{1}{2}\left(1+\ln\pi \right)∼1.07.$$

*$N_{S}$ is the minimum amount of ignorance displayed by Shannon’s entropy*. It could perhaps be thought of as a kind of information theory’s counterpart of the uncertainty ℏ/2 of quantum mechanics, although the units are different in the two cases. This least ℏ/2 amount of ignorance (with regards to canonically conjugate variables) is physically unavoidable, of course. the Shannon quantum entropic functional $S_{Q}$, instead, reflects an altogether distinct ignorance-amount (IA), that pertaining to the Born probability density ${\left|\psi \left(x\right)\right|}^{2}$. Can this IA be diminished if one chooses a different entropic measure? This is a seemingly interesting question, that will be answered in the affirmative in the next Section below. Let us make perfectly clear the following notion. A given minimum IA for an entropic functional (EF)

- in no way makes an EF “better” or “worse” than another EF,
- but it serves the purpose of classifying EFs using it and
- classification is the starting step of any scientific discipline [25].

### 4.2. Ignorance-Amount (IA) for Generalized Entropies

Our integrals over the variable *x* run always between −∞ and *∞*.

Tsallis’ entropy in the paradigmatic example [20]. In such case we will obtain a function $N_{T}\left(q\right)$ of *q* rather than a pure number. $N_{T}\left(q\right)$ depends on the specific value of the parameter *q* so that, after a straightforward computation, we get a real number $N_{T}$ for each *q* value. This real number arises from applying the super functional $F_{\alpha_{R}}$ to the functional $S_{Tq}\left[F_{\alpha_{R}}\right]$. Indeed,

(20) $$N_{T}\left(q\right)=F_{\alpha_{R}}\left(S_{Tq}\right)=S_{Tq}\left[F_{\alpha_{R}}\right]=\frac{1}{\sqrt{q}}\frac{\sqrt{q}-\pi^{\frac{1-q}{2}}}{q-1},$$

while, in Rényi’s case [20] we face the real numbers $N_{R}\left(q\right)$

(21) $$N_{R}\left(q\right)=F_{\alpha_{R}}\left(S_{Rq}\right)=S_{Rq}\left[F_{\alpha_{R}}\right]=\frac{1}{2(1-q)}\left[\ln\pi -\ln q-q\ln\pi \right]$$

Finally, for $N_{K}\left(q\right)$ - Kaniadakis, we find [22,23,24]

(22) $$N_{K}\left(q\right)=F_{\alpha_{R}}\left(S_{Kq}\right)=S_{Kq}\left[F_{\alpha_{R}}\right]=\frac{1}{2q}\left(\frac{1}{\pi^{\frac{q}{2}}}\frac{1}{\sqrt{1+q}}-\frac{1}{\pi^{-\frac{q}{2}}}\frac{1}{\sqrt{1-q}}\right).$$

The values of the super functional F are indeed independent of $\alpha_{R}$ and are all functions of π [and for all but Shannon’s, also of *q*]. the π-dependence comes, of course, from integrating a Gaussian function for the coherent states. We insist on the fact that we are facing here pure numbers. No physical units are involved.

If we carefully inspect the above equations, we will appreciate that, in some cases, the Shannon’s IA is diminished for the generalized functionals. This will be clearly seen in the graphs that we will display below.

### 4.3. Generalizing the $\alpha_{R}$-Independence to Arbitrary Entropic Measures

Let $G_{Q}$ be an arbitrary entropic measure that depends upon a set of parameters *Q* and involves the coherent-state probability density *F*, with $Q=(q_{1},q_{2},\ldots ,q_{n})$. We have the functional $F_{\alpha_{R}}\left(G_{C}\right)$

$$F_{\alpha_{R}}\left(G_{Q}\right)=\int G_{Q}\left[F_{\alpha_{R}}\left(x\right)\right]dx=$$

(23) $$\int G_{Q}\left[\pi^{-\frac{1}{2}}e^{-{\left(x-\sqrt{2}\alpha_{R}\right)}^{2}}\right]dx=\int G_{Q}\left[\pi^{-\frac{1}{2}}e^{-x^{2}}\right]dx=I_{Q}$$

and we see that the $\alpha_{R}$ dependence is gone, absorbed in a variables’ change that one makes in performing the Gaussian integrations, as above.

## 5. Results: Four Numerical Quantities Associated to Each of Our Monoparametric Ignorance Measures

**Figure 1:**
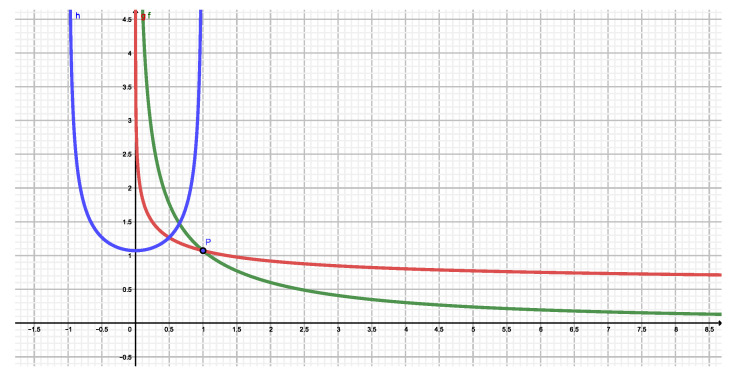
$N_{T}\left(q\right)$ versus *q*. The dark blue dot represents Shannon’s number $N_{S}$. the green curve corresponds Tsallis’ $N_{T}\left(q\right)$, the red one to Rényi’s $N_{R}\left(q\right)$, and the blue one is that associated to Kaniadakis’s $N_{K}\left(q\right)$.

These *N* quantities are (1) $N_{S}$, (2) $N_{T}\left(q\right)$, (3) $N_{R}\left(q\right)$, and (4) $N_{K}\left(q\right)$, associated respectively with Shannon, Tsallis, Rényi, and Kaniadakis. We plot and compare them. We see that Shannon’s ignorance amount can indeed be diminished by other entropic measures. Figure 1 one clearly demonstrates the fact that the Shannon’s ignorance amount is indeed decreased for q>1 in both the Tsallis and Rényi instances. Instead, Kanidakis’ functional achieves the same feat for *q* near zero.

**Figure 2:**
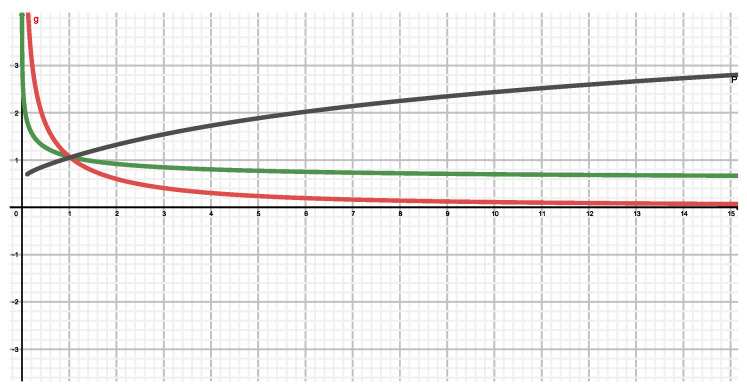
Two monoparametric functions N(q) versus *q*. Green for $N_{R}\left(q\right)$ and red for $N_{T}\left(q\right)$. the black curve displays $N_{R}\left(q\right)$ versus $N_{T}\left(q\right)$.

In Figure 2 we compare the ignorance amounts (IA) associated with Tsallis (horizontal) and Rényi (vertical) entropic forms.

The black curve displays $N_{R}\left(q\right)$ (vertical axis) versus $N_{T}\left(q\right)$ (horizontal one). A monotonic dependence is observed, as one should expect from the associated mathematical expressions for these entropic forms. the red curve tells us that Tsallis-IA is smaller than Rényi’s one for q>1. Viceversa for q<1.

**Figure 3:**
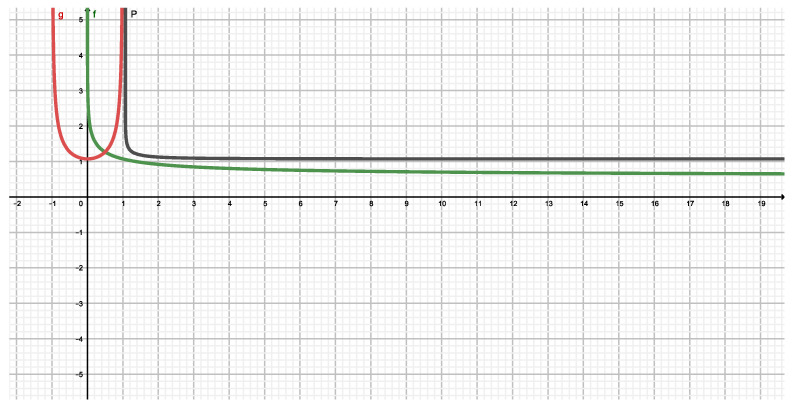
Two monoparametric functions N(q) versus *q*. Green for $N_{R}\left(q\right)$ and red for $N_{K}\left(q\right)$. the black curve displays $N_{R}\left(q\right)$ versus $N_{K}\left(q\right)$.

Figure 3 makes the comparisons as Figure 2, but now relates (black curve) Kaniadakis (vertical) to Rényi (horizontal) functionals. Here the black curve depicts the highly non trivial relationship between them.

## 6. Sharma-Mittal Biparametric Ignorance Measure

It is defined in term of two parameters *r* and *q* as [26,27]

(24) $$S_{SM_{\left(q,r\right)}}=\frac{1}{1-r}\left\{{\left\{\int {\left[F\left(x\right)\right]}^{q}dx\right\}}^{\frac{1-r}{1-q}}-1\right\},$$

so that

(25) $$F_{\alpha_{r}}\left(S_{SM_{\left(q,2q-1\right)}}\right)=\frac{1}{2-q}\left[{\left(\frac{\pi^{\frac{1-q}{2}}}{\sqrt{q}}\right)}^{\frac{2-q}{1-q}}-1\right],$$

where we have (arbitrarily, for comparisons ease) selected r=2q−1. For r=2 one has

(26) $$F_{\alpha_{r}}\left(S_{SM_{\left(q,2\right)}}\right)=1-\frac{1}{\sqrt{\pi }q^{\frac{1}{2q-1}}},$$

while for r=0.5 we have

(27) $$N_{SM}\left(q,r\right)=F_{\alpha_{r}}\left(S_{SM_{\left(q,0.5\right)}}\right)=2\pi^{\frac{1}{4}}q^{\frac{1}{4(q-1)}}-2.$$

**Figure 4:**
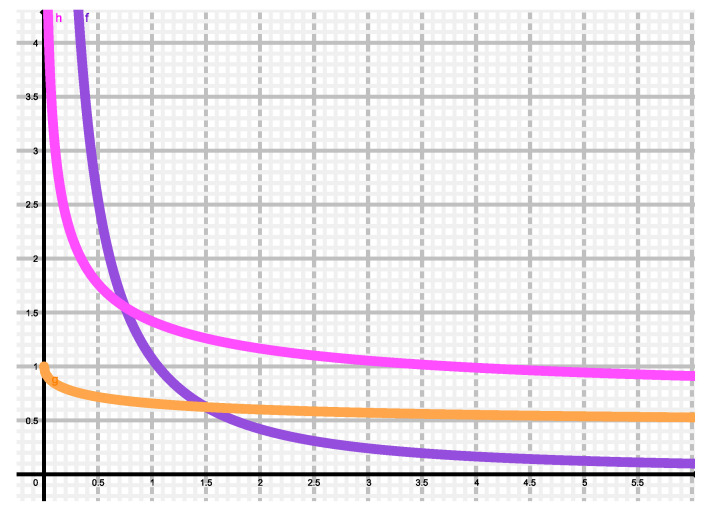
Sharma-Mittal’s $N_{SM}\left(q,r\right)=F_{\alpha_{R}}\left(S_{SM_{\left(q,r\right)}}\right)$ versus *q*. Purple for r=2q−1, orange for r=2, and purple for r=0.5.

The following graph (Figure 4) depicts our functional in terms of the pair (q,r).

**Figure 5:**
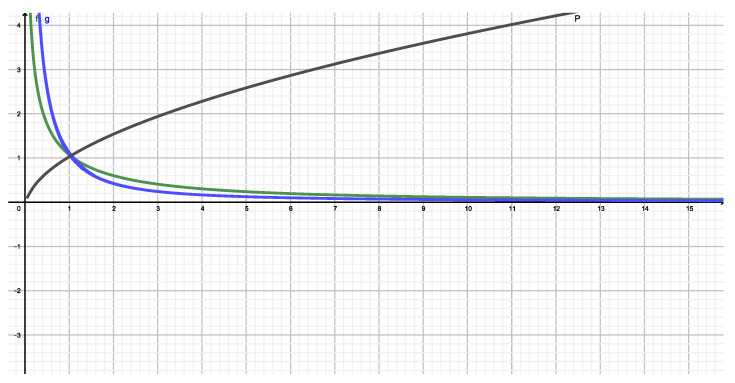
$F_{\alpha_{R}}$ is compared for (i) a monoparametric (Tsallis) versus (ii) a bi parametric one (Sharma-Mittal). the independent variable is *q*. the green curve represents $F_{\alpha_{R}}\left(S_{Tq}\right)$ while the blue one displays $F_{\alpha_{R}}\left(S_{SM_{\left(q,2q-1\right)}}\right)$ the black curve is different. It plots $F_{\alpha_{R}}\left(S_{Tq}\right)$ versus $F_{\alpha_{R}}\left(S_{SM_{\left(q,2q-1\right)}}\right)$.

The next figure (Figure 5) compares the Tsallis result to the Sharma-Mittal (q,2q−1) one.

We appreciate the fact that Sharma-Mittal measure exhibits a smaller ignorance amount than the Tsallis one for (0≤q≤∞). This is to be expected, since there are two free parameters.

## 7. Value of Our Dual Functional When the $S_{Q}$-Argument Is Not a Coherent State

For the sake of completeness, we show now that the numerical value *m* of $F[S_{Q}]$, when we deal with $S_{q}\left[F_{1}\right]$ (with $F_{1}$ the probability density for the HO first excited state), is larger than that for the same dual functional, when the argument of $S_{Q}$ is a coherent state.

This should lend credibility to the statement that coherent states’ information measures yield minimum values for the dual functional.

The expression for the first excited state wave function is

(28) $$\phi_{1}\left(x\right)=2x{\left(4\pi \right)}^{-\frac{1}{4}}e^{-\frac{x^{2}}{2}}.$$

Then,

(29) $$F_{\phi_{1}}\left(S\right)=-\frac{2}{\sqrt{\pi }}\int x^{2}e^{-x^{2}}\ln\left(\frac{2x^{2}}{\sqrt{\pi }}e^{-x^{2}}\right)dx,$$

so that (29) becomes

(30) $$F_{\phi_{1}}\left(S\right)=\frac{1}{2}\ln\pi +\ln2+C-\frac{1}{2}=m_{1}∼1.34,$$

where C=0.57721566490 is Euler’s constant. From (18) we see that $m_{1}>m\left(coherentstate\right)$.

## 8. Application to An Statistical Complexity (SC) Measure

Our entropy $S_{Q}$ can be viewed as the measure of the uncertainty associated to the basis-states on which the wave function (wf) is expanded (Cf. Equation (3)). We can regard the situation as that of a probabilistic physical processes described by the probability distribution $p_{j}={\left|c_{j}\right|}^{2};j=1;:::;N$, $P\equiv (p_{1};p_{2},\ldots ,p_{N})$, where *P* is a vector in a probability space. For $S_{Q}\left[P\right]=0$ the situation is that prevailing immediately after performing an experiment (wf “collapse” and minimum ignorance). On the other hand, our ignorance is maximal if S[P]=lnN (uniform probability). These two extreme circumstances of (i) maximum knowledge (or “perfect order”) and (ii) maximum ignorance (or maximum “randomness”) are regarded by many authors [1,2,3,4,5,6,7,8,9,10,11,28,29,30,31,32,33,34,35] as “trivial” ones. These authors have conceived the idea of devising a “measure” of the “statistical complexity” (SC) contained in *P* that would vanish in two extreme situations described above. We will analyze here, the quantum SC of which $S_{Q}$ is a basic ingredient. We will apply the quantifier *C* to the probability distribution (PD) $P=|\psi_{\alpha }{|}^{2}$ corresponding to coherent states. Accordingly, if C=0, the PD *P* would contain only trivial information. the larger *C*, the larger the amount of “non-triviality”. At this stage of our discussion emerges an important and well known observation. No all the available information measures are equally able to detect non-triviality. They are equally ‘informative.’ This is why we will analyze the PD *P* above with different C−measures, entailing distinct information measures (IM). Im turn. we study two different C−definitions.

### 8.1. Shiner-Davison-Landsberg Complexity Measure for Distinct IM

We appeal to the simplest SC measure $T_{SDL}$, devised by Shiner, Davison, and Landsberg (SDL) [36]. We first introduce the ratio *H* between $S_{Q}$ and the specific maximum value that $S_{Q}$ can attain ($S_{Q}^{maximal}$), that is,

(31) $$T_{SDL}=H\;\left(1-H\right).$$

**Figure 6:**
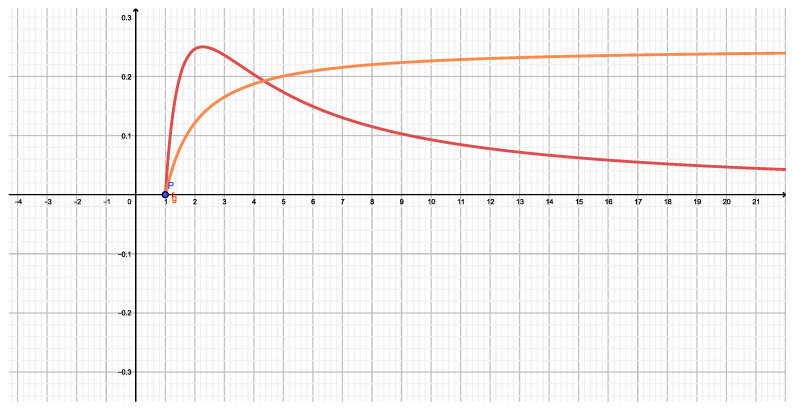
The Shiner-Davison-Landsberg complexity measure is plotted vs. *q* for Shannon, Tsallis, and Rényi entropic measures, as described in the text.

What are we looking at with this definition in our particular instance? Remember that here $P=|\psi_{\alpha }{|}^{2}$ corresponding to coherent states. But all our present entropic measures yield results that are independent of α as we have seen above. Thus, $T_{SDL}^{Shannon}=0$, not detecting any salient feature in *P*. Tsallis’ measure, instead, introduces another parameter, namely *q*, and correspondingly, $T_{SDL}^{Tsallis}\left(q\right)$ yields different values for different *q* and produces a q−parametrized curve- We plot in Figure 6 $T_{SDL}$ versus q∈[0,∞] for Shannon’s (q=1), Tsallis’ (red, q≥1) and Rényi’s (brown, q≥1) measures $S_{Q}^{q}$. As expected, the statistical complexity *T* vanishes at q=1, as we have just explained. For the q−entropies it grows first and then stabilize themselves. Tsallis-curve displays a maximum at q∼2.3, entailing a special q−value ∼2.3 of maximum complexity. What to we make of this maximum? that there are salient peculiarities in the distribution *P* above that the Tsallis SDL-measure best detect with this *q* value. the Rényi measure detection-ability grows with *q* at first, but eventually its non-triviality sensor stabilizes itself. Thus, if one is to apply *P* in computing some physical quantity *B*, the features of *B* should better be scrutinized via Tsallis’ measure with q=2.3, that would be the most “informative” one.

### 8.2. López Ruiz-Mancini-Calbet (LMC) Measure

The López Ruiz-Mancini-Calbet (LMC) is today regarded as the canonical SC measure, that has been applied to multiple physics-instances [28,29,30,31,32,33,34,35,37,38,39,40,41,42,43,44,45,46]. It has the following form:

(32) $$T_{LMC}=SQ,$$

where *Q* is called the disequilibrium and is a distance in probability space between the current probability distribution *P* and the uniform distribution. For continuous one-dimensional density probabilities *P* one has [1,2,3,4,5,6,7,8,9,10,11,28,29,30,31,32,33,34,35]

(33) $$Q=\int \;P^{2}dx.$$

**Figure 7:**
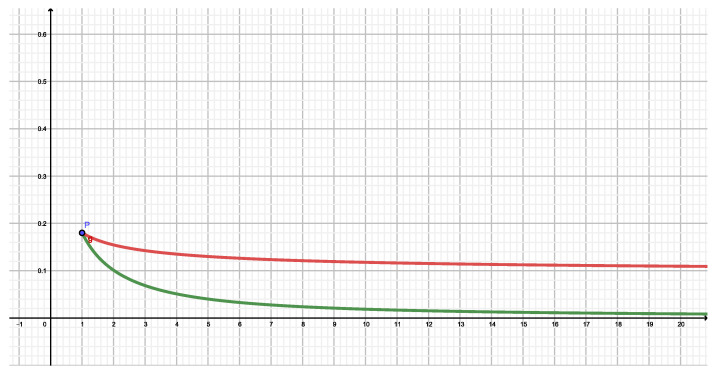
The López Ruiz-Mancini-Calbet complexity measure is plotted vs. *q* for Shannon, Tsallis, and Rényi entropic measures, as described in the text.

We have computed $T_{LMC}$ for the four probability distributions discussed above and plotted them versus *q* in Figure 7 Shannon blue dot, Tsallis green (q≥1) and Rényi red (q≥1). Note that no complexity maximum is displayed here by any of these curves. the LMC picture is the reverse of the SDL one. *C* is maximal for Shannon’s information measure, that becomes thus the most informative one. Rényi’s fares worse, and even more so Tsallis’. Moreover, in the two last cases the measures become less and less informative as *q* grows. Let us point out here that most people regard the LMC *C* as the canonical one, which has successfully detected phase transitions in many systems [28,29,30,31,32,33,34,37,38,39,40,41,42,43,44,45,46]. This, we construct our results as further evidence that LMC is better than SDL.

## 9. Conclusions

We have in this effort achieved a way of classifying the large number of different entropic functionals in vogue nowadays. This should be of importance in the sense of giving a semblance of order to the pandemonium of entropies galore that are used in a plethora of distinct scientific endeavors. Science always begins with a process of classification [25].

In our classification efforts we were aided by using the pure state entropy $S_{Q}$ advanced and utilized in References [9,10,11,12,13]. Our pure states are the coherent ones of the HO (CHO), taking advantage of the closed analytical representation of them advanced in References [18,19]. They are unique in the sense of possessing minimum Heisenberg uncertainty. We compute and compare diverse entropic functionals of the CHO probability densities.

Our quantum entropy $S_{Q}$ represents the information theoretic ignorance pertaining to the square modulus of ψ(x) when it is regarded as a probability density. As just stated, in this paper $\psi_{\alpha }\left(x\right)$ is an HO-coherent state, and for any entropic functional $S_{Q}$ one encounters a displacement-alpha independent, positive real value N(Q). This last fact gives sense to our central proposal, stated above, of associating to any entropic functional a numerical real value. N(Q) is the same for any arbitrary α and thus uniquely characterizes the entropic functional $S_{Q}$.

These numbers N(Q) provide a way of listing, and thus classifying, the plethora of extant literature’s entropic functionals. An application to statistical complexity measures (SCM) is made, that encounters significant differences between two popular SCM.
